# Oils with different degree of saturation: effects on ileal digestibility of fat and corresponding additivity and bacterial community in growing pigs

**DOI:** 10.1186/s40104-023-00990-6

**Published:** 2024-02-07

**Authors:** Lu Wang, Yifan Chen, Yuansen Yang, Nuo Xiao, Changhua Lai

**Affiliations:** 1 State Key Laboratory of Animal Nutrition, Ministry of Agriculture and Rural Affairs Feed Industry Centre, China Agricultural University, No. 2 Yuanmingyuan West Road, Haidian District, Beijing, 100193 China; 2 College of Animal Science and Technology, Hebei Agricultural University, Hebei, 071000 China

**Keywords:** Additivity, Bacterial community, Fat and fatty acids, Growing pigs, Ratio of unsaturated to saturated fatty acids, Standardized ileal digestibility

## Abstract

**Background:**

Oils are important sources of energy in pig diets. The combination of oils with different degree of saturation contributes to improve the utilization efficiency of the mixed oils and may reduce the cost of oil supplemented. An experiment was conducted to evaluate the effects of oils with different degree of saturation on the fat digestibility and corresponding additivity and bacterial community in growing pigs.

**Methods:**

Eighteen crossbred (Duroc × Landrace × Yorkshire) barrows (initial body weight: 29.3 ± 2.8 kg) were surgically fitted with a T-cannula in the distal ileum. The experimental diets included a fat-free basal diet and 5 oil-added diets. The 5 oil-added diets were formulated by adding 6% oil with different ratio of unsaturated to saturated fatty acids (U:S) to the basal diet. The 5 oils were palm oil (U:S = 1.2), canola oil (U:S = 12.0), and palm oil and canola oil were mixed in different proportions to prepare a combination of U:S of 2.5, 3.5 and 4.5, respectively.

**Results:**

The apparent and standardized ileal digestibility (AID and SID) of fat and fatty acids increased linearly (*P* < 0.05) as the U:S of dietary oils increased except for SID of fat and C18:2. The AID and SID of fat and fatty acids differed among the dietary treatments (*P* < 0.05) except for SID of unsaturated fatty acids (UFA) and C18:2. Fitted one-slope broken-line analyses for the SID of fat, saturated fatty acids (SFA) and UFA indicated that the breakpoint for U:S of oil was 4.14 (*R*^2^ = 0.89, *P* < 0.01), 2.91 (*R*^2^ = 0.98, *P* < 0.01) and 3.84 (*R*^2^ = 0.85, *P* < 0.01), respectively. The determined SID of fat, C18:1, C18:2 and UFA in the mixtures was not different from the calculated SID of fat, C18:1, C18:2 and UFA. However, the determined SID of C16:0, C18:0 and SFA in the mixtures were greater than the calculated SID values (*P* < 0.05). The abundance of *Romboutsia* and *Turicibacter* in pigs fed diet containing palm oil was greater than that in rapeseed oil treatment group, and the two bacteria were negatively correlated with SID of C16:0, C18:0 and SFA (*P* < 0.05).

**Conclusions:**

The optimal U:S for improving the utilization efficiency of mixed oil was 4.14. The SID of fat and UFA for palm oil and canola oil were additive in growing pigs, whereas the SID of SFA in the mixture of two oils was greater than the sum of the values of pure oils. Differences in fat digestibility caused by oils differing in degree of saturation has a significant impact on bacterial community in the foregut.

## Introduction

Oils are important source of energy in pig diets. In recent years, due to the rising costs of feed, there is consistent interest in maximizing the use of supplemental fat in the diet as nutritionists strive to increase the dietary energy density to meet the requirements of high-performing contemporary pigs [1]. The choice of oil to be used, under a given commercial condition, is largely driven by its cost. The combination of oils with different degree of saturation contributes to improve the utilization efficiency of the mixed oils and may reduce the cost of oil supplemented [2, 3]. In addition, the supplementation of oil with appropriate degree of saturation to the diets has the potential to improve pork quality [4].

The ratio of unsaturated to saturated fatty acids (U:S) of oil is one of the important determinants of the fat digestibility, and the U:S showed a high positive correlation with standardized ileal digestibility (SID) of fat [5]. However, the effect of U:S on the fat digestibility may not be linear [6]. In addition, the SID of fat and fatty acid can better reflect the availability of fat in pig diet [7]. Therefore, it is valuable to determine the optimal U:S of mixed oil based on the SID of fat and fatty acids for the effective utilization of oil in commercial practice.

Synergism, defined as the phenomenon whereby the dietary energy value of a saturated oil may be improved in the presence of a more unsaturated oil, was not in general detected [6, 8–10]. Fat digestibility of saturated oil and unsaturated oil is assumed to be additive without interactions, and thereby no synergism was observed in the mixed oils. However, no studies have been conducted to confirm this hypothesis.

Previous studies suggested that the type of fat is key to understanding the biological effects of high-fat diets on gut microbiome [11–13]. The fat digestibility varied greatly across oils with different degree of saturation [5]. Those indigestible fat may be a source of nutrients for bacteria and therefore have the potential to regulate gut bacterial community. Also, small intestine microbiota is important for host adaptive responses to the digestion and absorption of dietary lipids [14].

Therefore, the present experiment was conducted to: 1) evaluate the effect of oils with different U:S on the digestibility of fat and fatty acids of growing pigs to provide a reference for the mixing of oils in growing pigs; 2) test whether the digestibility of fat and fatty acids between saturated oil and unsaturated oil was additive; and 3) explore the effect of oils with different U:S on the bacterial community in the ileal digesta. The different U:S of oils were adjusted by a combination of palm oil and canola oil.

## Materials and methods

Ethics approval for the study was obtained from the Institutional Animal Care and Use Committee of China Agricultural University (Beijing, China; No. AW41103202-1-1). This experiment was conducted in the Metabolism Laboratory of Fengning Swine Research Unit of China Agricultural University (Chengde Jiuyun Agricultural and Livestock Co., Ltd., Hebei, China).

### Pigs and experimental diets

Eighteen crossbred (Duroc × Landrace × Yorkshire) barrows (initial body weight: 29.3 ± 2.8 kg) were housed individually in stainless steel metabolism crates (1.4 m × 0.7 m × 0.6 m) that allowed freedom of movement. Barrows were adapted to their environment for 7 d before surgery, and then simple T-cannulas were surgically implanted into each pig in the distal ileum as previously described by Stein et al. [15]. After a 10-day recovery from surgery, barrows were randomly allotted to 1 of 6 experimental diets in a 2-period Youden square design, which resulted in 6 observations per dietary treatment [16].

The palm oil and canola oil used in this experiment were food grade (free fatty acids < 0.2%). To determine the proportions of palm oil and canola oil in oil-mixing dietary treatment, the fatty acid compositions of palm oil and canola oil were determined before the animal trials (Table 1). The 6 diets included a fat-free basal diet and 5 oil-added diets (Table 2). The 5 oil-added diets were formulated by adding 6% oil with differing degree of fatty acid saturation to the basal diet at the expense of cornstarch. The 5 oils were palm oil (U:S = 1.2), canola oil (U:S = 12.0), and palm oil and canola oil were mixed in different proportions to prepare a combination of U:S of 2.5, 3.5 and 4.5, respectively. The same proportion of vitamins and minerals was supplemented across diets to meet or exceed the nutrient requirements of growing pigs as recommended by the NRC [17]. Diets contained 0.40% Cr_2_O_3_ as an indigestible marker to determine digestibility of fat and fatty acids. During the experimental period, the daily feed allowance was adjusted for each collection period on the basis of the pig’s body weight. All diets were stored at 4 °C to minimize fat oxidation and were allowed to adjust to room temperature at least 6 h before being fed.

**Table 1:** Fatty acid composition of canola oil and palm oil, % (of total fatty acids)

Fatty acid	Canola oil	Palm oil
C10:0	0.08	0.18
C12:0	0.10	1.94
C14:0	0.43	8.54
C15:0	0.17	0.38
C16:0	35.85	342.82
C16:1	1.86	1.46
C17:0	0.40	0.82
C18:0	15.91	40.21
C18:1n9c	480.19	369.11
C18:2n6c	164.99	99.96
C18:3n3	78.47	1.58
C20:0	5.26	3.43
C20:1	16.81	1.30
C21:0	1.10	0.05
C20:4n6	0.03	−
C20:3n3	0.08	−
C22:0	2.92	0.62
C22:1n9	17.77	0.04
C23:0	0.22	0.14
C24:0	1.26	0.67
C24:1	1.64	−
SFA	63.71	399.80
UFA	761.85	473.45
U:S	11.96	1.18

“ − ” Below the limit of quantification

*SFA* Saturated fatty acid, *UFA* Unsaturated fatty acid, *U:S* The ratio of unsaturated to saturated fatty acids

**Table 2:** Composition (%, as-fed basis) and chemical analysis (%, dry matter basis) of experimental diets

Item	Fat-free diet	Ratio of unsaturated to saturated fatty acids				
		1.2	2.5	3.5	4.5	12.0
Ingredients						
Cornstarch	44.00	38.00	38.00	38.00	38.00	38.00
Soy protein isolate	15.50	15.50	15.50	15.50	15.50	15.50
Canola oil	0.00	0.00	2.76	3.76	4.39	6.00
Palm oil	0.00	6.00	3.24	2.24	1.61	0.00
Sucrose	10.00	10.00	10.00	10.00	10.00	10.00
Sugar beet pulp	26.00	26.00	26.00	26.00	26.00	26.00
Dicalcium phosphate	2.00	2.00	2.00	2.00	2.00	2.00
Limestone	0.70	0.20	0.20	0.20	0.20	0.20
Sodium chloride	0.40	0.40	0.40	0.40	0.40	0.40
Chromic oxide	0.40	0.40	0.40	0.40	0.40	0.40
Vitamin-mineral premix^1^	0.50	0.50	0.50	0.50	0.50	0.50
L-Lys HCl, 78.8%	0.20	0.20	0.20	0.20	0.20	0.20
DL-Met, 98.5%	0.12	0.12	0.12	0.12	0.12	0.12
L-Thr, 98.5%	0.18	0.18	0.18	0.18	0.18	0.18
Analyzed composition						
Dry matter	88.67	89.32	89.39	89.59	89.40	89.58
Acid-hydrolyzed ether extract	0.63	6.31	5.63	6.36	6.76	6.33
Crude protein	20.06	19.38	19.84	19.40	19.38	19.13
Neutral detergent fiber	11.04	12.14	11.64	11.34	12.01	11.99
Acid detergent fiber	6.19	6.24	6.20	6.08	5.32	6.25
Calculated composition						
Metabolizable energy, MJ/kg	13.96	15.11	15.06	15.04	15.01	14.93
Ratio of unsaturated to saturated fatty acids	−	1.18	2.47	3.46	4.45	11.96
Calcium	0.99	0.99	0.99	0.99	0.99	0.99
Available phosphorus	0.39	0.39	0.39	0.39	0.39	0.39

^1^Vitamin-mineral premix provided the following per kg of complete diet for growing pigs: vitamin A, 5,512 IU; vitamin D_3_, 2,200 IU; vitamin E, 30 IU; vitamin K_3_, 2.2 mg; vitamin B_12_, 27.6 μg; riboflavin, 4.0 mg; pantothenic acid, 14.0 mg; niacin, 30.0 mg; choline chloride, 400.0 mg; folacin, 0.7 mg; thiamine 1.5 mg; pyridoxine, 3.0 mg; biotin, 44.0 ug; Mn, 40.0 mg (MnO); Fe, 75.0 mg (FeSO_4_**·**H_2_O); Zn, 50.0 mg (ZnO); Cu, 15.0 mg (CuSO_4_**·**5H_2_O); I, 0.3 mg (KI); Se, 0.3 mg (Na_2_SeO_3_)

### Feeding and sample collection

Barrows were weighed at the beginning of each period and supplied with experimental diets at 4% of their body weight. Diets were given each day as two equal meals at 08:00 and 17:00 h. Fresh water was available at all times. Room temperature was maintained at 22 ± 2 °C. Humidity varied from 55% to 65%.

Each experimental period lasted 8 d. Barrows were adapted to experimental diets for 6 d followed sequentially by 2-d collection of ileal digesta. Freshly ileal digesta samples were collected as previously described [7] and immediately stored at –20 °C. In addition, freshly ileal digesta samples were also collected using a 5-mL sterilized plastic tube and then immediately placed in liquid nitrogen, and stored in a freezer at −80 °C for further analysis.

### Sample preparation and analyses

Before analyses, ileal digesta samples were thawed and pooled for each pig within experimental period, and a subsample was collected. Digesta subsamples were lyophilized in a vacuum-freeze dryer (Tofflon Freeze Drying Systems, Shanghai, China) and ground through a 1-mm screen.

The fatty acid profiles of the palm oil and canola oil were determined using Gas Chromatography (6890 Series, Agilent Technologies, Wilmington, DE, USA) following a modification of the procedures of Sukhija and Palmquist [18]. Experimental diets were analyzed for dry matter (DM; method 930.15) [19], crude protein (CP; method 990.03) [19], acid-hydrolyzed fat (AEE; method 954.02) [19], neutral detergent fiber (NDF) and acid detergent fiber (ADF), fatty acid profiles and chromium. The content of ADF and NDF were determined using F57 filter bags and fiber analyzer equipment (Fiber Analyzer; Ankom Technology, Macedon, NY, USA) according to the procedure of van Soest et al. [20] with the slight modification. The NDF was analyzed using heat stable α-amylase and sodium sulfite without correction for insoluble ash. The chromium concentration was determined using a polarized Zeeman Atomic Absorption Spectrometer (Hitachi Z2000, Tokyo, Japan) after nitric acid-perchloric acid wet ash sample preparation. Ileal digesta samples were analyzed for DM, AEE, fatty acid profiles and chromium.

Ileal digesta samples stored at –80 °C were used for the bacterial community analysis. In the present study, three dietary treatments with U:S of 1.2, 3.5 and 12.0 could be used to represent the high, middle, and low degree of saturation groups, respectively. Therefore, the bacterial community analysis was not conducted for the remaining treatments as it was considered unnecessary and would have added complexity to the interpretation of the results. The DNA kit (Omega Bio-tek, Norcross, GA, USA) was used for bacterial DNA extraction according to the manufacturer’s instructions. NanoDrop 2000 UV–VIS spectrophotometer (Thermo Scientific, Wilmington, DE, USA) was used to determine DNA concentrations. The 1% agarose gel electrophoresis proved that DNA isolation was achieved as expected. The V3–V4 hypervariable regions of the bacteria 16S rRNA gene were amplified with primers 338F (5′-ACTCCTACGGGAGGCAGCAG-3′) and 806R (5′-GGACTACHVGGGTWTCTAAT-3′) by thermocycler PCR system (GeneAmp 9700, ABI, Foster City, CA, USA). The PCR products were extracted from a 2% agarose gel and then purified using the AxyPrep DNA Gel Extraction Kit (Axygen Biosciences, Union City, CA, USA) and quantified using QuantiFluor™-ST (Promega, Madison, WI, USA). Pooled and purified amplicons in equimolar and paired-end were sequenced (2 × 300 bp) on an Illumina MiSeq platform (Illumina, San Diego, CA, USA) according to the standard protocols introduced by Majorbio Bio-pharm Technology Co., Ltd. (Shanghai, China). The original files were quality-filtered by Trimmomatic (version 3.29) and merged by FLASH. Operational taxonomic units (OTUs) were clustered with 97% similarity cutoff using UPARSE (version 7.1 http://drive5.com/uparse/). The taxonomy of each 16S rRNA gene sequence was analyzed by Ribosomal Database Project Classifier algorithm against the Silva (SSU123) 16S rRNA database using confidence threshold of 90%.

### Calculation

The basal endogenous losses of fat and fatty acids were calculated from pigs fed the fat-free diet as previously described by Wang et al. [5]. The apparent ileal digestibility (AID) and the SID of fat and fatty acids were calculated from analyzed concentrations of fat and fatty acids and markers in diets and ileal digesta [5].

The calculated SID of fat in the mixture of palm oil and canola oil were derived with the following equation:

$$\begin{array}{c}\mathrm{SID_{CM}}=\mathrm{SID_{P}}\times \mathrm{C_{P}}+\mathrm{SID_{R}}\times \mathrm{C_{R}},\\ \mathrm{C_{P}}=\mathrm{FC_{P}}\times \mathrm{P_{P}}/ (\mathrm{FC_{P}}\times \mathrm{P_{P}}+\mathrm{FC_{R}}\times \mathrm{P_{R}}),\\ \mathrm{C_{R}}=\mathrm{FC_{R}}\times \mathrm{P_{R}}/ (\mathrm{FC_{P}}\times \mathrm{P_{P}}+\mathrm{FC_{R}}\times \mathrm{P_{R}}),\end{array}$$

in which C_P_ and C_R_ were the fat contribution coefficients of palm oil and canola oil to their mixture, respectively. The FC_P_ and FC_R_ were the fat concentrations of palm oil and canola oil, respectively. The P_P_ and P_R_ were the proportions of palm oil and canola oil in oil-mixing dietary treatment, respectively. The SID_CM_ (%) was the calculated SID of fat in the mixture of palm oil and canola oil, and SID_P_ and SID_R_ represented the determined SID of fat in palm oil and canola oil, respectively. The SID_CM_ of saturated fatty acids (SFA) and unsaturated fatty acids (UFA) were calculated using the equation shown above.

### Statistical analyses

All data were analyzed statistically using the MIXED procedure of SAS (version 9.4; SAS Institute Inc., Cary, NC, USA). Homogeneity of variance was verified using the UNIVARIATE procedure of SAS. Dietary treatment was a fixed effect and pig and period were the random terms. Individual pig was the experimental unit for all analyses. The LSMEANS procedure was used to calculate mean values of all dietary treatments. Orthogonal polynomial contrasts were used to determine the linear and quadratic effects of increasing U:S of oils on AID and SID of fat and fatty acids. The SID of fat and fatty acids were estimated by a one-slope broken-line model using NLIN procedure of SAS to determine the break point for the optimal U:S. The *t*-test procedure was used to compare the differences between the calculated and the determined values for SID of fat and fatty in the mixture of palm oil and canola oil.

The R software (version 3.3.1) was used to analyze the bacterial diversity, and Kruskal–Wallis test was used to compare the relative abundance of bacteria in each group at phylum, family and genus level. The linear discriminant analysis effect size (LEfSe) analysis combined with an all-against-all multi-group comparison strategy was used to compare differences in taxonomic levels, including phylum, class, order, family and genus. The logarithmic linear discriminant analysis value of 2.0 was used to be the criterion. The comparative analysis between U:S 1.2 treatment and U:S 12.0 treatment was conducted based on the method of Wilcoxon rank sum test. The correlations between bacterial abundance (at the genus level) and digestibility of fat and fatty acids were analyzed by Spearman correlation analysis. The greater the absolute value of the correlation coefficient, the stronger the correlation. For all analyses, the statistical significance if *P* < 0.05 and considered a trend if 0.05 < *P* < 0.10.

## Results

All pigs remained in good health and ileal digesta samples were successfully obtained from all pigs during the collection period.

### Ileal digestibility of fat and fatty acids

Data on the AID and SID of fat and fatty acids of oils differing in degree of saturation fed to growing pigs were presented in Tables 3 and 4, respectively. The AID and SID of fat and fatty acids increased linearly (*P* < 0.05) as the U:S of dietary oils increased except for SID of fat and C18:2. The AID and SID of fatty acids increased quadratically (*P* < 0.05) as the U:S of dietary oils increased except for SID of C18:2 and UFA. The AID and SID of fat had a tendency to increased quadratically (*P* = 0.09) as the U:S of dietary oils increased. The AID and SID of fat and fatty acids differed among the dietary treatments (*P* < 0.05) except for SID of UFA and C18:2. The AID and SID of fat for canola oil were greater than that of palm oil (*P* < 0.05).

**Table 3:** Apparent ileal digestibility of fat and fatty acids of oils differing in degree of saturation fed to growing pigs, %

Item	Ratio of unsaturated to saturated fatty acids					SEM	*P*-value		
	1.2	2.5	3.5	4.5	12.0		ANOVA	Linear	Quadratic
Fat	73.8^b^	74.9^ab^	81.6^ab^	81.3^ab^	85.0^a^	2.47	0.02	< 0.01	0.09
C16:0	71.6^b^	86.1^a^	88.3^a^	89.8^a^	89.3^a^	2.58	< 0.01	0.03	< 0.01
C18:0	62.2^b^	78.1^a^	82.4^a^	86.3^a^	86.9^a^	2.95	< 0.01	< 0.01	< 0.01
C18:1	94.3^b^	96.0^ab^	96.3^ab^	96.9^a^	97.5^a^	0.60	0.01	< 0.01	0.03
C18:2	90.5^b^	93.7^ab^	93.6^ab^	94.9^a^	95.7^a^	0.90	0.01	< 0.01	0.02
SFA	69.9^b^	83.7^a^	85.5^a^	87.1^a^	88.4^a^	2.59	< 0.01	0.02	< 0.01
UFA	93.1^b^	95.1^ab^	95.4^ab^	96.2^a^	96.8^a^	0.69	0.01	< 0.01	0.02

*SFA* Saturated fatty acid, *UFA* Unsaturated fatty acids

^a,b^Within a line, values without a common superscript differ (*P* < 0.05). *n* = 6

**Table 4:** Standardized ileal digestibility of fat and fatty acids of oils differing in degree of saturation fed to growing pigs, %

Item	Ratio of unsaturated to saturated fatty acids					SEM	*P*-value		
	1.2	2.5	3.5	4.5	12.0		ANOVA	Linear	Quadratic
Fat	87.9^b^	90.6^ab^	95.5^ab^	95.4^ab^	98.6^a^	2.46	0.04	0.07	0.09
C16:0	74.5^b^	91.0^a^	94.8^a^	95.5^a^	98.7^a^	2.56	< 0.01	< 0.01	< 0.01
C18:0	69.2^b^	87.8^a^	93.7^a^	93.7^a^	97.4^a^	2.95	< 0.01	< 0.01	< 0.01
C18:1	95.3^b^	96.9^ab^	97.1^ab^	97.7^ab^	98.3^a^	0.59	0.02	< 0.01	0.04
C18:2	96.8	98.5	98.1	99.2	99.7	0.90	0.24	0.06	0.25
SFA	74.0^b^	90.3^a^	94.1^a^	95.7^a^	97.8^a^	2.38	< 0.01	< 0.01	< 0.01
UFA	95.8	97.2	98.4	98.0	99.6	1.07	0.24	0.04	0.41

*SFA* Saturated fatty acid, *UFA* Unsaturated fatty acids

^a,b^Within a line, values without a common superscript differ (*P* < 0.05). *n* = 6

**Fig. 1:**
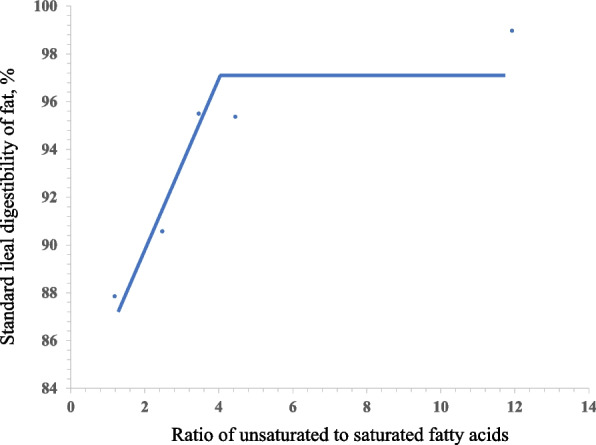
Fitted broken-line of the ratio of unsaturated to saturated fatty acids (U:S) of oil versus standard ileal digestibility (SID) of fat. Each data point represents least squares means of 6 observations. Each regression model shows the SID of fat (*y*) relative to U:S (*x*). The linear broken-line model for the SID of fat indicated that the breakpoint for U:S of oil was 4.14 based on the following equation: *y* = 97.17 – 3.30 × (4.14 – *x*) where *x* is less than 4.14 (*R*^2^ = 0.89, *P* < 0.01)

**Fig. 2:**
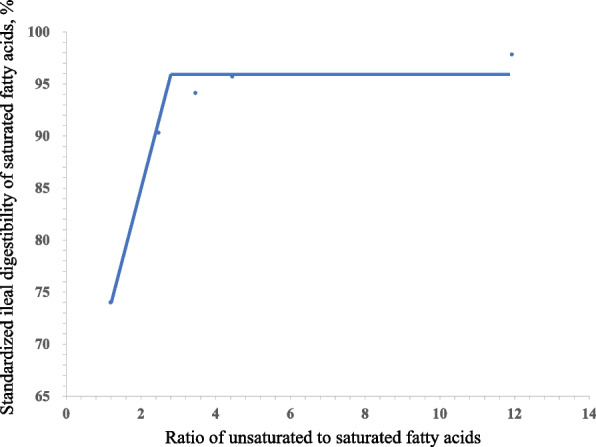
Fitted broken-line of the ratio of unsaturated to saturated fatty acids (U:S) of oil versus standard ileal digestibility (SID) of saturated fatty acids (SFA). Each data point represents least squares means of 6 observations. Each regression model shows the SID of SFA (*y*) relative to U:S (*x*). The linear broken-line model for the SID of SFA indicated that the breakpoint for U:S of oil was 4.14 based on the following equation: *y* = 95.90 – 12.65 × (2.91 –* x*) where *x* is less than 2.91 (*R*^2^ = 0.98, *P* < 0.01)

**Fig. 3:**
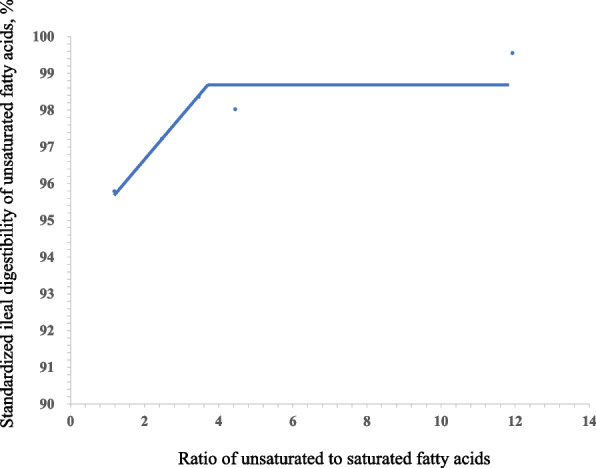
Fitted broken-line of the ratio of unsaturated to saturated fatty acids (U:S) of oil versus standard ileal digestibility (SID) of unsaturated fatty acids (UFA). Each data point represents least squares means of 6 observations. Each regression model shows the SID of UFA (*y*) relative to U:S (*x*). The linear broken-line model for the SID of UFA indicated that the breakpoint for U:S of oil was 3.84 based on the following equation: *y* = 98.88 – 1.14 × (3.84 – *x*) where *x* is less than 3.84 (*R*^2^ = 0.85, *P* < 0.01)

Fitted one-slope broken-line analyses (2 straight-line and one-breakpoint model) for the SID of fat, SFA and UFA indicated that the breakpoint for U:S of oil was 4.14 (*R*^2^ = 0.89, *P* < 0.01), 2.91 (*R*^2^ = 0.98, *P* < 0.01) and 3.84 (*R*^2^ = 0.85, *P* < 0.01) (Fig. 1, 2 and 3).

### Additivity of digestibility of oils differing in degree of saturation

The SID of fat and fatty acids of the mixtures calculated from the SID values of the two pure oils (palm oil and canola oil) and their respective proportions in the mixtures were compared with determined values. Results indicated that the determined SID of fat, C18:1, C18:2 and UFA in the mixtures was not different from the calculated SID of fat, C18:1, C18:2 and UFA (Table 5). However, the determined SID of C16:0, C18:0 and SFA in the mixtures were greater than the calculated SID values (*P* < 0.05).

**Table 5:** Calculated and determined values for standardized ileal digestibility of fat and fatty acids in mixtures of palm oil and canola oil^1, 2^

Item	Mixture 1		SEM	*P-*value	Mixture 2		SEM	*P*-value	Mixture 3		SEM	*P*-value
	Calculated values	Determined values			Calculated values	Determined values			Calculated values	Determined values		
Fat	92.8	90.6	2.88	0.473	94.6	95.5	4.19	0.838	95.7	95.4	0.96	0.725
C16:0	76.5	91.0	1.58	< 0.001	78.1	94.8	1.24	< 0.001	79.9	95.5	1.17	< 0.001
C18:0	76.4	87.8	1.70	0.001	80.5	93.7	2.15	0.002	83.8	93.7	1.45	0.001
C18:1	96.9	96.9	0.56	0.954	97.4	97.1	0.47	0.660	97.7	97.7	0.37	0.901
C18:2	98.5	98.5	0.62	0.969	98.9	98.1	0.57	0.194	99.2	99.2	0.66	0.982
SFA	76.9	90.3	1.47	< 0.001	79.2	94.1	1.35	< 0.001	81.4	95.7	1.50	< 0.001
UFA	98.0	97.2	0.60	0.255	98.6	98.4	0.38	0.101	98.9	98.0	0.47	0.132

*SFA* Saturated fatty acid, *UFA* Unsaturated fatty acids

^1^Mixtures 1, 2 and 3 were palm oil and canola oil mixed in different proportions to produce mixture with U:S of 2.5, 3.5 and 4.5, respectively

^2^Calculated value is the sum of the standardized ileal digestibility value of a single oil times the rate contributed by the single oil in mixture. *n* = 6

### Bacterial community in ileal digesta

**Fig. 4:**
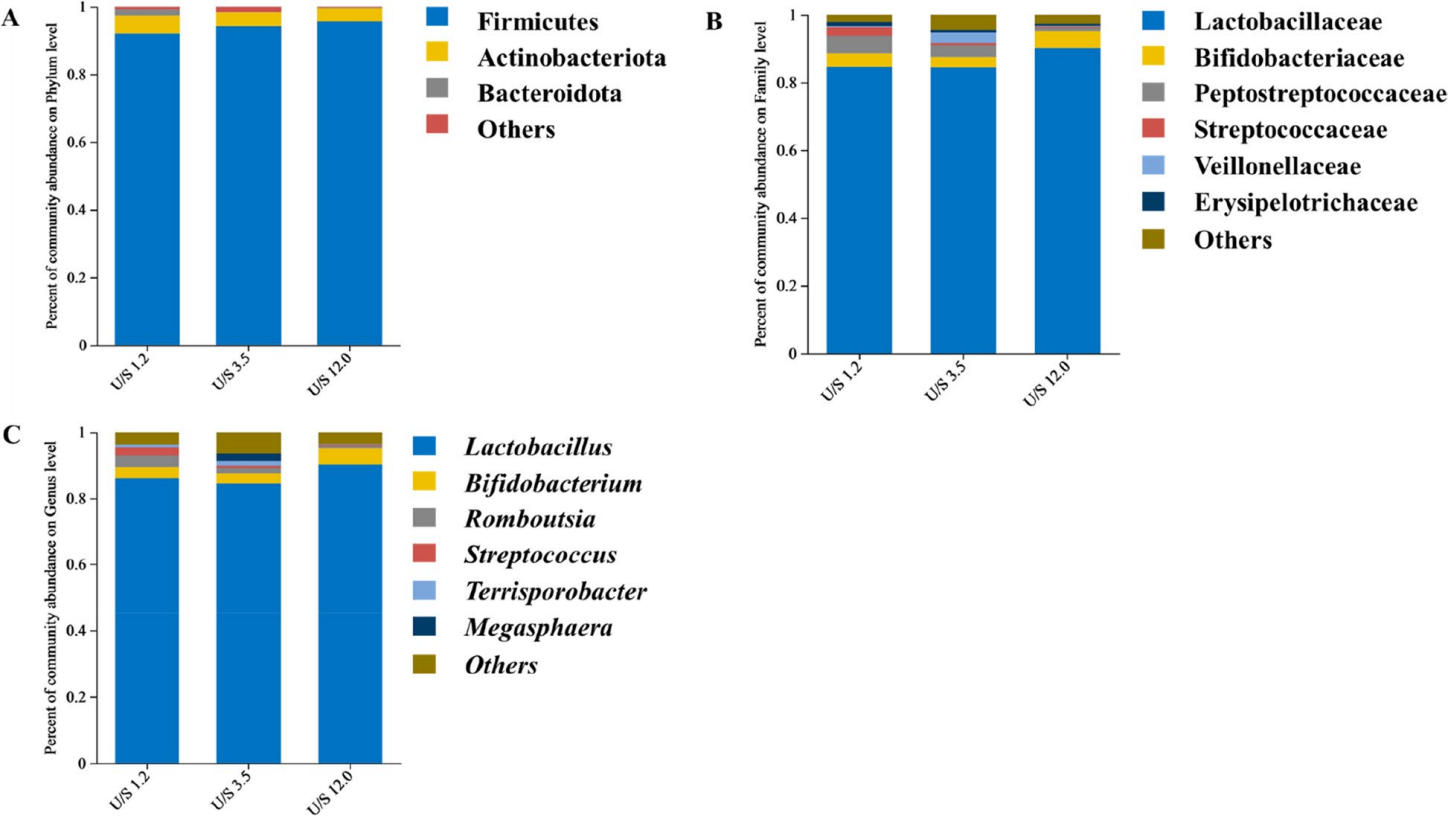
Bacterial community structure at the phylum (**A**), family (**B**) and genus (**C**) levels in growing pigs. Phyla, families, and genera with proportions less than 1% are not listed. *U:S* Ratio of unsaturated to saturated fatty acids. *n* = 6

**Fig. 5:**
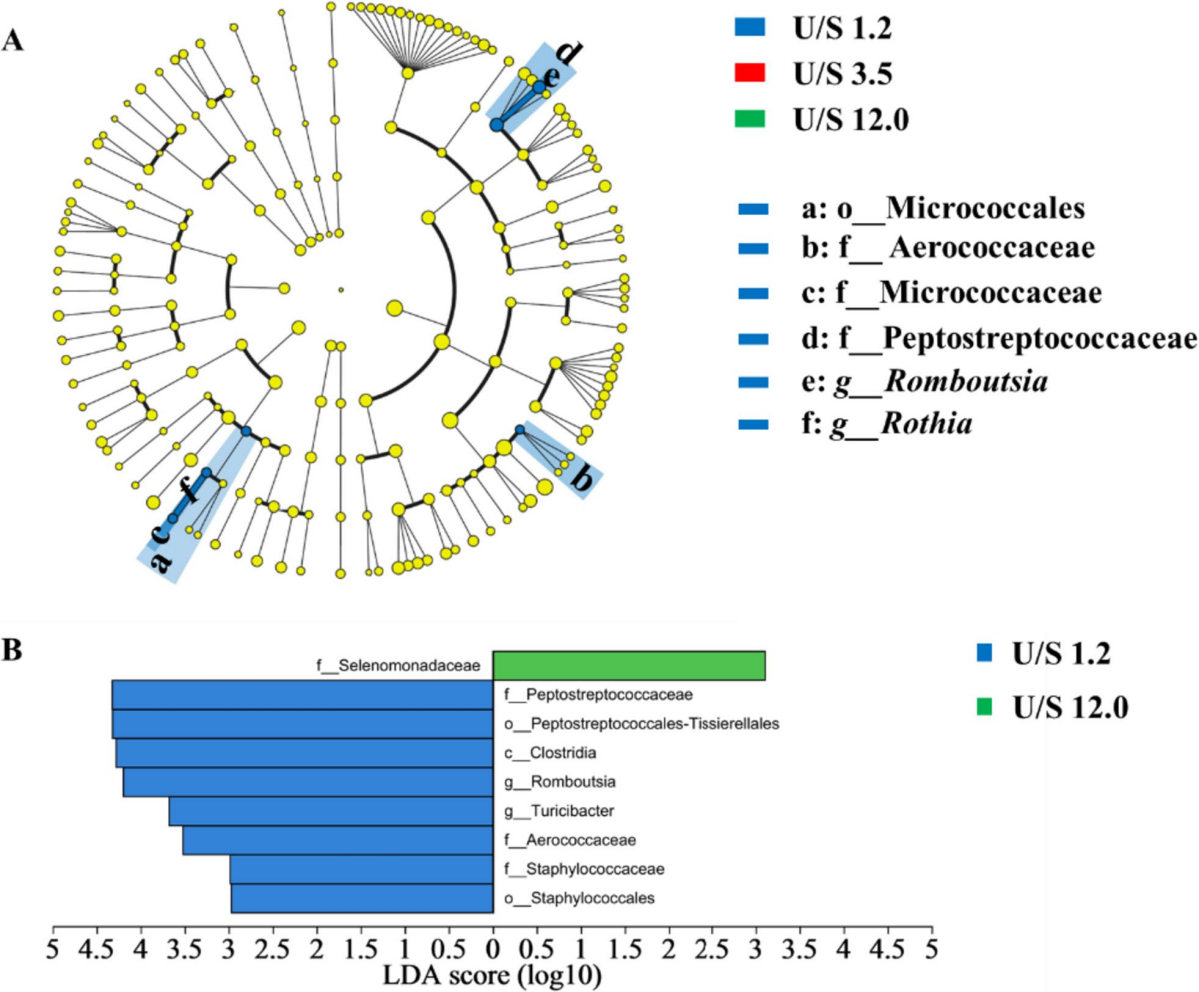
The linear discriminant analysis effect size results of the bacterial community. Histogram of the linear discriminant analysis scores computed for the differentially abundant features across U:S 1.2, U:S 3.5 and 12.0 treatments (**A**) and between U:S 1.2 and 12.0 treatments (**B**). *U:S* Ratio of unsaturated to saturated fatty acids.* n* = 6

The indices of Shannon, Simpson, Ace and Chao at the OTU level were used to elevate bacterial richness and diversity. There was no significant difference in α diversity of bacterial community among treatments (Table 6). A phylum-level analysis proved that the microbiota composition in ileal of pigs was consistently dominated by Firmicutes and Actinobacteriota, accounting for 94% and 4%, respectively (Fig. 4A). Down to the family level, the predominant bacteria were Lactobacillaceae (86%), Bifidobacteriaceae (4%) and Peptostreptococcaceae (3%) were the dominant bacteria (Fig. 4B). At the genus level, the dominant genera were *Lactobacillus*, *Romboutsia*, *Bifidobacterium* and *Streptococcus* (Fig. 4C)*.* The specific bacterial taxa associated with treatments was identified by LEfSe (LDA score > 2) analysis. The results showed 6 different bacterial taxa across three treatments (Fig. 5A). A large abundance of Micrococcales, Aerococcaceae, Micrococcaceae, Peptostreptococcaceae, *Romboutsia* and *Rothia* in the dietary treatments with U:S of 1.2 were detected. Compared with the U:S 12.0 treatment, the U:S 1.2 treatment had an increased abundance of Peptostreptococcaceae, Peptostreptococcales-Tissierellales, Clostridia, *Romboutsia*, *Turicibacter*, Aerococcaceae, Staphylococcaceae and Staphylococcales and a decreased abundance of Selenomonadaceae (Fig. 5B).

**Table 6:** The α diversity of bacterial community in ileal digesta

Item	U:S 1.2	U:S 3.5	U:S 12.0	*P-*value
Shannon	1.79	1.75	1.85	0.832
Simpson	0.29	0.26	0.26	0.740
Ace	103.69	101.13	103.12	0.832
Chao	101.61	94.00	100.33	0.894

*U:S* Ratio of unsaturated to saturated fatty acids. *n* = 6

### Correlations between bacterial abundance with digestibility of fat and fatty acids

**Fig. 6:**
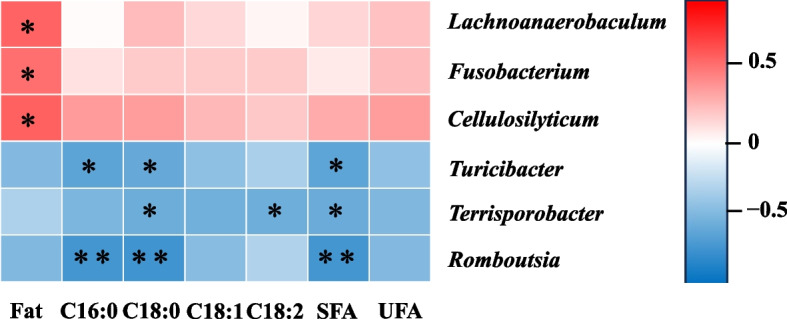
Heatmap of Spearman’s correlations between bacterial abundance (at the genus level) and digestibility of fat and fatty acids. *U:S* Ratio of unsaturated to saturated fatty acids. ^*^P < 0.05; ^**^P < 0.01. *n* = 6

For differential bacteria at the genus level (Fig. 6), the abundance of *Lachnoanaerobaculum, Fusobacterium, *and* Cellulosilyticum* was positively correlated with SID of fat (*P* < 0.05). The abundance of *Turicibacter *and* Romboutsia* was negatively correlated with SID of C16:0, C18:0, and SFA (*P* < 0.05). In addition, the abundance of *Terrisporobacter* was negatively correlated with SID of C18:0, C18:2, and SFA (*P* < 0.05).

## Discussion

Added oil was the sole source of fat and fatty acids in the oil-added diets. Therefore, the AID and SID for fat and fatty acids in the test diet were equal to that of the oil. In addition, only free-forms (extracted oils) were used in this work and the digestibility values were comparable with the reference data [21]. The unsaturated oil (canola oil) had a greater digestibility value than that of the saturated oil (palm oil). This observation agrees with results showing a greater digestibility of canola oil versus palm oil in growing pigs [5, 22, 23] and weaned pigs [23].

Both AID and SID of fat and fatty acids increased curvilinearly as U:S of dietary oils increased. This is due to the oils with a higher proportion of UFA will have a higher digestibility and oils with a low U:S will have a reduced digestibility [5, 24]. There are many reasons why UFA are easier to digest than SFA, including 1) UFA have the greater ability to infiltrate the bile salt micelle compared with SFA [25, 26]; 2) pancreatic lipase appears to have a greater affinity for polyunsaturated fatty acids than SFA [27, 28]; and 3) a low molecular weight binding proteins of the intestinal mucosa may preferentially bind UFA over SFA [29]. Similarly, a previous study reported that apparent digestibility of individual fatty acids increases with increasing unsaturation [30]. However, there is a report that no differences in the apparent digestibility of fat were observed when pigs fed diets containing 3% fat with U:S ratio of 3, 4 or 5 [31]. This may be due to the relatively high minimum U:S (= 3) and narrow range of U:S (3−5) in the research of Incharoen et al. [31]. Studies have shown that a less rapid increase in fat digestibility when the U:S was greater than 2.08 [6, 8].

It has been confirmed that U:S is positively related to the SID of fat [5]. The result of the present experiment indicated that the SID of fat increased with an increasing U:S. However, the effect was larger at a lower U:S (< 3.5) than at a greater one. When U:S increased from 1.2 to 3.5, the average increase for SID of fat was 3.30% for one point increase in the U:S, but from 3.9 to 12.0 the average increase was only 0.36%. This result reflects the fact that the effect of U:S of dietary oil on the utilization of fat is not linear. In contrast, the increase in fat utilization leveled off with the U:S of oil increased. These findings were in agreement with that of Wiseman et al. [6] who reported that a considerable improvement in the digestibility of fat occur when the U:S increases from 0.93 to 2.08, but thereafter the increase was less dramatic. In addition, a similar trend was observed with digestible energy values of soybean oil, tallow and their blends or canola oil, palm oil (or tallow) and their blends when given to growing/finishing pigs [6, 8].

Standardized digestibility is a more accurate estimate of fatty acid bioavailability than apparent digestibility [7]. Thus, broken-line models for SID of fat and fatty acids were fitted. In determining SID values, a key issue is an estimation of the endogenous fat losses. Our previous study reported that the fat-free diet method can accurately estimate values for SID of fat and fatty acids [7]. In the present experiment, basal endogenous losses of fat and fatty acids were similar to our previous results in growing pigs [5].

From the point of cost reduction and practicality, blends of saturated oil and unsaturated oil represent an economic option. However, the optimal U:S for mixed oils has not been determined. In the present experiment, oils of different degrees of saturation were blended in fixed proportions to give mixed oil varying in U:S. A one-slope broken-line analyses of the SID indicated that the SID of fat, SFA and UFA entered a plateau when the U:S of oil were 4.14, 2.91 and 3.84 respectively. After that, the increase of SID values would be small as the U:S of oil increased. Therefore, the present experiment suggested that optimal U:S for improving the utilization efficiency of mixed oil was 4.14. Incharoen et al. [31] reported that dietary fat with U:S of 4 was optimal for growing pigs based on energy utilization, which was similar to our finding. In addition, considering that the digestibility of SFA is lower than that of UFA [32, 33], it is suggested that the minimum U:S of 2.91 was required for growing pigs to facilitate the absorption of SFA from mixed oil. This result was consistent with a previous report from Doppenberg and van der Aar [2], which suggested that a minimum U:S of 2.25 is required for growing pigs to prevent a lower digestible energy than 98% of the maximum. Therefore, in commercial practice, oil sources with a high content of SFA, such as palm oil or tallow oil, should not be used as a single oil source. This means that the oil with a high content of UFA such as soybean oil also needs to be supplemented to improve the efficiency of dietary fat utilization. Notably, when adjusting the U:S in the compound feed, the fatty acid composition of intact oil in the basal diet should also be considered. Furthermore, the extent of difference in digestion and absorption between SFA and UFA may depend on age of animals: young pigs may require the greater U:S in the diet to improve the efficiency of fat utilization compared with growing/finishing pigs [10, 34, 35]. Thus, further studies are needed to determine the optimal and minimum U:S of oil in the diet for young pigs.

The present study is the first to determine whether the SID of fat in mixed oils are additive for growing pigs. The approach adopted in the present study, by blending oils of extreme U:S to generate mixtures with intermediary U:S, allowed an assessment of the additivity of values for digestibility of fat and fatty acids in mixtures. Such an analysis revealed that there were no differences between determined and calculated SID of fat or UFA in mixtures. Accordingly, the SID of fat and UFA for palm oil and canola oil were additive. It can be concluded that the SID of fat or UFA in mixtures can be predicted from the value for the two separate oils.

The determined energy value of a mixture containing two oils, one that is relatively saturated and the other one that is relatively unsaturated, may be greater than that calculated from the numerical values obtained for the two separate oil sources. This interaction is often referred to as the phenomenon of ‘synergism’ (also known as ‘associative effects’) [6]. However, evidence to support ‘synergism’ is not strong. In fact, numerous studies in pigs have shown that synergism between oils was not in general detected [6, 8, 10]. The present study indicated that the SID of fat for saturated oil will not increase in the presence of a more unsaturated oil, which may explain why synergism was not observed in the mixed oils. Therefore, the synergism between oils is conceptually unsound. However, the synergism between individual fatty acids differing in degree of saturation is accepted. The current data demonstrated that the determined values for SID of SFA in the mixtures were greater compared with the calculated SID values. This result confirms that the absorption of SFA can be enhanced by supplementing UFA. The SFA alone have a lower micellar formation potential and thus are less efficiently digested than UFA. The presence of UFA or monoglycerides can improve the efficiency of micelle formation and contribute to subsequent absorption of SFA [1, 25, 32]. Such synergism has been reported in the chick, where it was observed that oleic acid and linoleic acid can effectively increase the absorption of free palmitic acid and stearic acid, and oleic acid appears to play a direct role in facilitating the absorption of the SFA [36]. Similarly, Freeman [25] reported that oleic acid is as effective as monoglycerides in increasing the solubility of stearic acid. Therefore, the SID of SFA for palm oil and canola oil were not additive in mixed oils fed to growing pigs.

Responses of the microbiota to dietary intervention can be rapid, with changes observed within 1 d [37, 38]. In this study, a short-term intervention was sufficient to cause a shift in gut microbes. Because different types of dietary fat have great impact on gut microbial composition in mammals [11, 12] and the digestion and absorption of fat is mainly in the foregut [7], we explored the effects of three oils differing in degree of saturation on the microbial community in the ileal digesta of growing pigs. No significant differences in the bacteria abundance at the phylum level were observed, but changes in *Romboutsia* and *Rothia* at the genus level were detected. The pathogenicity of *Romboutsia* had not yet to be confirmed [39, 40]. Zhu et al. [41] reported that abundance of *Romboutsia* may be negatively correlated with fat absorption in the jejunum under high-fat conditions. This is consistent with our research results that *Romboutsia* was enriched in the palm oil treatment with lowest digestibility of fat and fatty acids. In addition, palm oil treatment group is rich in *Rothia*. The genus *Rothia* has been reported to be an opportunistic pathogen associated with various infections [42], which may also contribute to the decreased digestibility of fat and fatty acids in pigs fed palm oil diets. The relative abundance of Selenomonadaceae was greater in canola oil treatment group compared with palm oil treatment group. There are few studies on the structural changes of Selenomonadaceae and its clinical and physiological significance in humans and pigs. Previous studies showed that adding Selenohomolanthionine [43] or probiotic product [44] to the diets of goats or dogs increased the relative abundance of the family Selenomonadaceae.

The digestion and absorption of dietary fat were influenced by the small intestine microbiota [12, 14]. The abundance of *Lachnoanaerobaculum, Fusobacterium, *and* Cellulosilyticum* was positively correlated with SID of fat. Hedberg et al. [45] isolated *Lachnoanaerobaculum* from the human small intestine and found that butyrate was one of the main metabolites of *Lachnoanaerobaculum*. Butyrate can provide nutrients for the tissue and protect the integrity of the intestinal mucosa [46], which contribute to the digestion and absorption of nutrients. It is well known that *Cellulosilyticum* is a cellulose-degrading bacteria [47]. The results of this study suggest that it may also be related to fat digestion. In addition, the abundance of *Turicibacter, Terrisporobacter, *and* Romboutsia* was negatively correlated with SID of SFA. Wan et al. [48] reported that increased palm stearin intake significantly expanded the relative abundance of *Turicibacter.* In the present study, compared with the canola oil treatment, the palm oil treatment with lower digestibility of fat and fatty acids had higher abundance of *Turicibacter*, which may lead to a negative correlation between the *Turicibacter* and digestibility of SFA. *Terrisporobacter*, an anaerobic pathogen [49], has been proven to induce oxidative stress [50]. As mentioned before, *Romboutsia* may be negatively correlated with fat absorption in the jejunum under high-fat conditions [41]. These findings provide evidence that oils with different degree of saturation can affect fat digestibility and further influence composition of small intestine microflora.

## Conclusion

The present experiment suggested that optimal U:S for improving the utilization efficiency of mixed oil was 4.14, and the minimum U:S was 2.91. The SID of fat and UFA for palm oil and canola oil were additive without interactions in growing pigs, whereas the SID of SFA in the mixture of two oils was greater than the sum of the values of pure oils. In addition, differences in fat digestibility caused by oils differing in degree of saturation has a significant impact on bacteria community in the foregut. The abundance of *Romboutsia* and *Turicibacter* in pigs fed diet containing palm oil was greater than that in rapeseed oil treatment group, and the two bacteria were negatively correlated with SID of C16:0, C18:0 and SFA.

## Data Availability

The data analyzed during the current research are available from the corresponding author on reasonable request.

## Abbreviations

ADF: Acid detergent fiber
AEE: Acid-hydrolyzed fat
AID: Apparent ileal digestibility
CP: Crude protein
DM: Dry matter
LEfSe: The linear discriminant analysis effect size
NDF: Neutral detergent fiber
NRC: National Research Council
OUT: Operational taxonomic units
PCR: Polymerase chain reaction
SFA: Saturated fatty acids
SID: Standardized ileal digestibility
U:S: The ratio of unsaturated to saturated fatty acids
UFA: Unsaturated fatty acids
